# 

**Published:** 1889-01

**Authors:** 


					﻿Minnesota State Homoeopathic Society.—The Twenty-third Annual.
Meeting of the Minnesota State Homoeopathic Institute will be held at St.
Paul, May 21st to 23d. A full and useful meeting is expected. The pro-
gramme of the work to be done gives promise of this.
NOTICE.
All articles for publication, books for review, business communications, eto.,
must be sent to Editors, 1123 Spruce Street, Philadelphia.
Subscribers will please notice that this Journal will be sent until arrears
are paid and it 'is ordered discontinued.
NOTICE TO SUBSCRIBERS.
The price of THE HOMOEOPATHIC PHYSICIAN for
this year will be as before, $2.50; to those who pay before
April 1st, the price will be only $2.00. A prompt remit-
tance will, therefore, save you 50 cents, and help us.
The amount must be sent directly to this office, and not
through any newspaper or other agency, in order to secure
the reduction.
				

